# Establishing cell polarity in plants: the role of cytoskeletal structures and regulatory pathways

**DOI:** 10.3389/fcell.2025.1602463

**Published:** 2025-05-09

**Authors:** Chao Ma, Jianbin Chang, Olivia S. Hazelwood, M. Arif Ashraf, Qiong Nan

**Affiliations:** ^1^ Institute of Future Agriculture, Northwest A&F University, Xianyang, Shaanxi, China; ^2^ Department of Botany, University of British Columbia, Vancouver, BC, Canada

**Keywords:** cell polarity, actin, microtubule, polarity proteins, polarity regulation

## Abstract

Cell polarity is a fundamental mechanism of plant cells that drives cellular specialization and the formation of diverse cell types. It regulates critical developmental events, including polarized tip growth (such as pollen tubes and root hairs), epidermal patterning (such as trichome branching and asymmetric cell division in stomata). The establishment and maintenance of cell polarity rely on the cytoskeleton-mediated polarized distribution of specific proteins and organelles. In particular, cell-type-specific actin and microtubule dynamic structures are pivotal for maintaining polarity. For example, actin cables and short actin fragments are critical for pollen tube growth, while actin clusters and microtubule rings are involved in trichome branching, and actin patches contribute to stomatal mother cell polarization. Beyond directing the polarization of organelles and proteins, the cytoskeleton itself serves as an intrinsic cue for polarity. For instance, actin patches in stomatal precursor cells act as self-organizing polarity landmarks. Despite the diversity of cytoskeletal structures and their functions, common regulators, such as Rop GTPase signaling pathways, WAVE/SCAR complexes, and motor proteins regulate the assembly and function of these structures. Recent advances have revealed new regulatory mechanisms, such as microtubule exclusion zones guiding asymmetric divisions during Arabidopsis stomatal development, and the role of actin rings in regulating xylem pit formation. These discoveries contribute to a deeper understanding of the cytoskeleton’s crucial role in polarity regulation. In this review, we highlight the key cytoskeletal structures involved in the establishment of cell polarity in plants and discuss the molecular mechanisms underlying their spatiotemporal assembly. We also address emerging questions regarding the cytoskeleton’s role in cell polarity and development.

## Introduction

Cell polarity is essential for cellular function and multicellular development (Muroyama and Bergmann, 2019; Zhang and Dong, 2018). It arises from the subcellular asymmetric distribution of cell components, such as asymmetrically localized proteins and organelles, ultimately forming cell-type-specific morphological polarity (Qi and Greb, 2017). In plants, as sessile organisms, cell polarity is crucial for the formation of various cell types. A prime example of this is found in tip-growing cell types, such as pollen tubes, root hairs, and Physcomitrium patens protonemata (Muroyama and Bergmann, 2019; Yang, 2008). In these cells, growth is restricted to a small distal area, creating a tip at the apex of the elongating cell. Cell polarity also needs to be established in diffusely growing cell types, such as trichomes (Bouyer et al., 2001; Yang, 2008) and during the morphogenesis of pavement cell lobes (Yang, 2008). This process relies on spatially coordinated polarity signals that locally regulate the establishment of cell polarity.

Cell polarity is a critical prerequisite for asymmetric cell division, a fundamental mechanism underlying cellular differentiation and diversity. Prior to asymmetric division, cells must establish polarity, which can be influenced by two factors: intrinsic polarity inherited from the mother cell or extrinsic signals received before division. Asymmetric cell division results in two daughter cells with distinct fates (De Smet and Beeckman, 2011; Muroyama and Bergmann, 2019), as observed in the development of stomata and zygotes. Furthermore, cell polarity is essential for polar transport processes, which facilitate the asymmetric distribution of signaling molecules such as the plant hormone auxin, thereby guiding plant development (Naramoto, 2017).

In plants, as in other eukaryotes, the cytoskeleton—comprising actin filaments (AFs) and microtubules (MTs)—is essential for establishing cell polarity. Both AFs and MTs participate in various critical cellular functions (Smith, 2003). Their ability to rapidly assemble and disassemble enables quick, localized coordination in response to the establishment of cell polarity.

Pharmacological and genetic studies, combined with cytoskeletal imaging techniques, have shown that specific cytoskeletal structures play crucial roles in cell-type specification and cell polarization. Depolymerization of cytoskeletal structures leads to detectable impairments in cell-type specification. One example is pollen tube growth, where actin cables are found in the shank and short actin fragments are present at the tips of pollen tubes in Arabidopsis. The reassembly of AFs following drug-induced or mutation-induced depolymerization blocks tip growth and disrupts cell polarity, demonstrating that the cytoskeleton is necessary for cell polarization (Gibbon et al., 1999). Similarly, MTs exhibit distinct arrangements during pollen tube tip growth. In pollen tubes, MTs are absent from the tip but form longitudinal bundles in older regions of the tube. These MTs play key roles in endocytosis, exocytosis, and the transport of reproductive nuclei during polar growth (Idilli et al., 2013). Other cell types, such as trichomes (Tian et al., 2015), moss protonemata (Wu and Bezanilla, 2018), Arabidopsis zygotes (Kimata et al., 2016), and stomatal formation, also depend on specific cytoskeletal structures for proper development (Facette et al., 2015).

Recent studies have revealed novel dynamic changes in cytoskeletal arrangements across diverse plant cell types, where specific arrangements of AFs and MTs mediate specialized cellular formations (Sugiyama et al., 2019). In Arabidopsis xylem pit formation, for instance, where the actin cytoskeleton forms a ring-like structure, directing localized cell wall modifications (Sugiyama et al., 2019; Kijima et al., 2025). In Arabidopsis stomatal mother cells, MTs exhibit localized depolymerization to create “clear zones”, enabling asymmetric division (Muroyama et al., 2023). During rice pollen germination, AFs and MTs form radial patterns emanating from the germination aperture. Notably, some cell types exhibit cytoskeletal elements that self-organize into polarized aggregates, as seen in rice pollen germination (Liu et al., 2022), while other cell types lack intrinsic polarity but establish polarized domains through interactions with polarity proteins or organelles (Denninger et al., 2019; Gendre et al., 2019). These configurations enable the coordination of morphogenetic processes by providing mechanical force, acting as tracks for organelle movement, facilitating endo/exocytosis at the polarity site, and forming polarity cues through polarized aggregation. Through such mechanisms, cytoskeletal dynamics establish and maintain cellular polarity, ultimately dictating the geometry of tip growth and other shape-determining events.

Although cytoskeletal structures may vary in their arrangement and shape across different cell types, they often share common regulatory factors that modulate their dynamics through related signaling pathways. Several cytoskeletal regulatory proteins, including actin-related protein-2/3 (Arp2/3), ROP-interactive CRIB motif-containing (RIC), and kinesin-like calmodulin-binding (KCBP), act at cell polarity sites to regulate cytoskeleton dynamics and reorganization. Additionally, upstream signals have been shown to precisely recruit and activate these cytoskeletal regulators in a spatially and temporally coordinated manner, such as through the ROP GTPase pathway, the WAVE-Arp2/3 pathway, and myosin/kinesin motor (Le et al., 2006; Wasteneys and Galway, 2003). Notably, polarity proteins can directly or indirectly influence the actin cytoskeleton at the polarity site, thereby regulating cell type formation. For example, in Arabidopsis, the polarity crescent defined by BREAKING OF ASYMMETRY IN THE STOMATAL LINEAGE (BASL) and BREVIS RADIX family (BRXf) proteins controls stomatal development, while in maize, the polarity protein WEAK CHLOROPLAST MOVEMENT UNDER BLUE LIGHT 1 (WEB1)/PLASTID MOVEMENT IMPAIRED 2 (PMI2)-RELATEDA/B (WPRA/B) regulates the polarized accumulation of actin patches in subsidiary guard cells (Muroyama et al., 2023; Nan et al., 2022).

In this review, we highlight specialized cytoskeletal architectures involved in plant cell polarity and their associated functions. We also discuss molecular regulators that control cytoskeletal dynamics and how they integrate with upstream signaling pathways. By synthesizing recent advances in the field, we address key questions regarding the relationship between cell polarity and the cytoskeleton.

## Cytoskeleton organization and functional roles in polarity across plant cell types

The cytoskeleton exhibits a dynamic organization in plant cells, playing a critical role in the regulation of cell morphology and function, particularly in processes related to cell polarity. Research on cytoskeletal contributions to cell polarity has primarily focused on two aspects: structural organization and functional roles (Wasteneys and Galway, 2003). Various model systems, including pollen tubes, root hairs, trichomes, pavement cells, moss protonemal cells, and cells undergoing asymmetric division, have provided insights into the cell type-specific arrangements of AFs and MTs. These specialized cytoskeletal structures are crucial for establishing and maintaining cell polarity and play key roles in regulating cellular growth and differentiation.

### Cytoskeletal organization and roles in tip growth

Tip growth in plants represents an extreme form of polar growth, where growth is confined to the apex or apical dome, leading to a cylindrical cell shape (Rounds and Bezanilla, 2013). This growth pattern is observed in structures such as pollen tubes, root hairs, and moss protonemata. Tip growth is highly dependent on the cytoskeleton, which is essential for maintaining cell polarity and directing growth.

**FIGURE 1:**
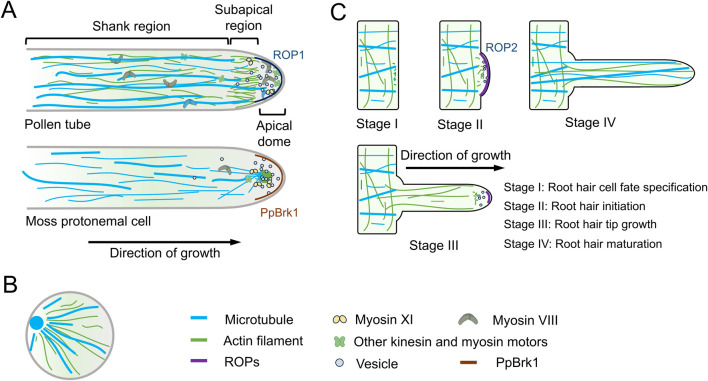
Dynamics of actin filaments and microtubules in tip growth. **(A)** In pollen tubes, actin filaments (AFs) and microtubules (MTs) exhibit distinct organizational dynamics in different regions. These specific cytoskeletal arrangements enable pollen tubes to coordinate vesicle transport, cell wall formation, and the rapid establishment of polarity during tip growth. Moss protonemal cells, on the other hand, possess a unique cytoskeletal organization, further emphasizing the diversity of cytoskeletal architectures among different tip-growing cell types. Additionally, the polar localization of ROP1/PpBrk1 plays a critical role in tip growth. **(B)** During rice pollen germination, both AFs and MTs form radial patterns radiating from the germination pore. **(C)** Root hairs represent another form of tip growth. A core function of AFs in root hairs is transporting membrane compartments to the root tip, thereby delivering cell wall components and membrane lipids required for apical growth. MTs play a crucial role in maintaining the directional growth of root hairs. In growing Arabidopsis root hairs, ROP2 localizes to the root hair tip.

In pollen tubes, AFs are organized into at least three distinct structural configurations (Figure 1A). In the shank, AFs are arranged axially into bundles exhibiting uniform polarity. At the subapex, AFs form regular structures, known as the collar, fringe, mesh, or funnel, depending on the species of pollen tube (Qu et al., 2015; Rounds and Bezanilla, 2013). In the apical region, AFs are shorter, less abundant, and highly dynamic. It has been proposed that these distinct AF structures perform specialized functions during polar growth of the pollen tube. In the shank, AFs facilitate the transport of organelles and vesicles from the base to the tip along the cell cortex. At the subapex, cytoplasmic streaming reverses direction, with the flow moving toward the base along the axial actin cables located at the center of the tube, generating a reverse-fountain pattern of cytoplasmic streaming. In the apical region, AFs play multiple roles, including regulating vesicle trafficking, preventing retrograde vesicle movement, and driving tip-directed vesicle transport (Qu et al., 2015; Rounds et al., 2014; Xiang et al., 2007; Ye et al., 2009). These specific cytoskeletal arrangements enable the pollen tube to coordinate vesicle transport, cell wall formation, and the rapid establishment of polarity during tip growth.

MTs are not present at the tip of the pollen tube but are instead organized into longitudinally aligned bundles in the older sections of the tube, located around 25–30 μm from the tip’s plasma membrane. In both the shank and the apex, MTs are arranged as short, randomly oriented strands (Idilli et al., 2013) (Figure 1A). While disrupting MTs generally has little effect on tip growth in many angiosperm pollen tubes (Cai and Cresti, 2010), it has been shown to influence endocytosis and exocytosis at the tip of tobacco (Nicotiana tabacum) pollen tubes (Idilli et al., 2013). Recent research suggests that MTs are crucial for the proper positioning and polar movement of the generative nucleus in Arabidopsis pollen, which is vital for the successful delivery of sperm cells during double fertilization (Wang et al., 2024). These findings highlight the specialized structural arrangements and functions of MTs in pollen tube growth.

Recent studies on rice pollen grain germination have revealed novel cytoskeletal arrangements, where both AFs and MTs form radial patterns that radiate from the germination aperture (Figure 1B). Furthermore, actin bundles and MTs interact and co-function during pollen grain germination, this arrangemnet and dynamic is specific in rice pollen aperture, though the precise regulatory mechanisms remain unclear (Liu et al., 2022).

Root hairs represent another form of tip growth that shares similarities with pollen tubes in terms of growth mechanisms. In Arabidopsis root hairs, AFs initially accumulate at a bulging region (Figure 1C), marking the future position of the tip-growing root hair (Ishida et al., 2008). During growth, an irregular actin mesh forms in the subapical region, with highly dynamic fine filaments extending into the root hair apex (Era et al., 2009) (Figure 1C). A central function of AFs in root hairs is to deliver membrane compartments to the apex, providing the necessary cell wall components and membrane lipids required for tip growth (Ishida et al., 2008). However, the precise mechanisms underlying these processes remain an area of active investigation.

MTs in root hairs are organized into dense cortical microtubules (CMTs), which are longitudinally arranged along the shank of the root hair (Figure 1C). Notably, MTs are absent from the apex of growing root hairs (Figure 1C); however, in fully developed root hairs, longitudinally oriented MTs can extend into the tip region (Van Bruaene et al., 2004) (Figure 1C). Pharmacological assays involving the microtubule-depolymerizing drug oryzalin and the microtubule-stabilizing drug taxol have demonstrated that disruption of MTs results in a loss of directionality in Arabidopsis root hair growth. This finding suggests that MTs play a crucial role in maintaining the directional growth of root hairs (Bibikova et al., 1999). However, the precise mechanisms by which MTs regulate root hair polarity remain to be fully elucidated.

Moss protonemal cells provide an excellent model system for studying tip growth, with a distinct cytoskeletal organization compared to pollen tubes and root hairs (Rensing et al., 2020). In the tip of protonemal cells, AFs accumulate in an apical cluster (Figure 1A), with the intensity and position of this cluster changing dynamically during tip growth (Finka et al., 2007; Furt et al., 2013). Notably, this apical actin cluster resembles the fungal Spitzenkörper—a multicomponent pleomorphic structure critical for maintaining hyphal tip growth, which contains chitosomes, ribosomes, AFs, and MTs. However, whether these two structures are evolutionarily related remains unknown.

Cytoplasmic MTs in protonemal cells are aligned longitudinally throughout the cytoplasm and converge at the apex of the growing cell (Wu and Bezanilla, 2018) (Figure 1A). This unique cytoskeletal arrangement in moss protonemal cells further highlights the diversity of cytoskeletal organization across different tip-growing cells.

The apical actin cluster in moss protonemal cells spatially overlaps with myosin XI and secretory vesicles (Finka et al., 2007; Vidali et al., 2009; Vidali et al., 2010) (Figure 1A), indicating that this actin cluster is involved in polarized secretion and contributes to the determination of growth direction. This suggests that the actin cluster plays a critical role in guiding the directional growth of protonemal cells (Wu and Bezanilla, 2018). In contrast, the role of MTs in this process remains less understood. Pharmacological inhibition of MTs disrupts the directionality of protonemal cell growth but does not completely abolish tip growth (Doonan et al., 1988). Further research indicates that the apical convergence of MTs is crucial for the formation of the actin cluster at the cell tip, highlighting the interdependence of these cytoskeletal systems in the regulation of tip growth (Wu and Bezanilla, 2018). The distinct organization of AFs and MTs in moss protonemal cells offers valuable insights into newly discovered mechanisms that regulate polarity growth.

### Cytoskeletal organization and roles in diffuse growth cell types

In addition to tip growth, the cytoskeleton plays a critical role in establishing cell polarity in anisotropically growing cell types, such as trichomes and epidermal pavement cells in Arabidopsis (Bouyer et al., 2001; Fu et al., 2005; Muroyama and Bergmann, 2019). These cell types increase their surface area diffusely while differentially expanding specific regions or positions within the cell.

**FIGURE 2:**
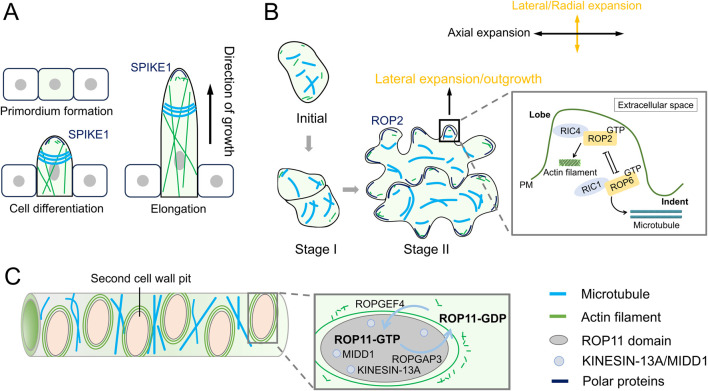
Dynamics of actin filaments and microtubules during diffuse growth. **(A)** During the formation of trichomes in Arabidopsis thaliana, actin filaments (AFs) form an actin network at the tip, while microtubules (MTs) form transverse rings. **(B)** In the formation of pavement lobes, AFs and MTs also exhibit distinct dynamic distributions. Cortical fine AFs localize to the top of the lobe outgrowth, whereas MTs are arranged transversely in the neck region of lobes. In pavement cells (PCs), two antagonistic ROP pathways (ROP2- and ROP6-GTPase pathways) cooperatively regulate lobe morphogenesis. The ROP2-RIC4 interaction drives lobe outgrowth by promoting actin assembly, whereas the ROP6-RIC1 interaction restricts lobe expansion through microtubule organization. PM, plasma membrane. **(C)** During pit formation in xylem cells, AFs and MTs regulate cell wall deposition as well as the size and aperture of pits. ROP domains coordinate secondary cell wall patterning. Active ROP11 (activated by ROPGEF4/ROPGAP3) recruits MIDD1 and kinesin-13A to pit domains, creating microtubule-free zones that suppress wall deposition.

In Arabidopsis trichome branching, a key feature of the actin cytoskeleton is the tip-localized actin meshwork found within the microtubule-depleted zone of the trichome branch. This microtubule-depleted zone is located at the extreme apex of the elongating branch and is surrounded by transverse rings that exhibit a typical tip-directed microtubule (MT) density gradient (Tian et al., 2015; Yanagisawa et al., 2015) (Figure 2A). The actin meshwork is essential for maintaining cell wall thickness gradients, which are necessary for the tip-biased diffuse growth of the trichome branch (Yanagisawa et al., 2015). The MT rings are believed to play a crucial role in determining the branch position during trichome development (Sambade et al., 2014).

Another model for studying diffuse growth in plants is the formation of pavement lobes, which requires finely tuned coordination between the actin and MT cytoskeletons. During Arabidopsis lobe formation, cortical fine AFs localize to the top of the lobe outgrowth, where well-ordered cortical MTs are absent. Meanwhile, MTs are arranged transversely in the neck region of the lobe (Wasteneys and Galway, 2003) (Figure 2B). These transversely arranged MTs restrict expansion in the direction of their predominant orientation, while the fine AFs facilitate lobe initiation and outgrowth (Fu et al., 2005).

Xylem cells serve as the primary channel for nutrient and water transport in plants, and their pitted cell walls (small openings in the walls) play a crucial role in facilitating efficient water movement. The formation of pits is tightly regulated by actin polarity and the establishment of a MT clear zone. During pit formation, the MT clear zone creates a pit domain that is devoid of microtubules, thereby inhibiting cell wall deposition within this region. In contrast, MTs outside the pit domain are stabilized (Figure 2C), contributing to proper cell wall formation. Additionally, AFs form rings around the pit (Figure 2C), regulating both cell wall deposition and the size and aperture of the pit (Sugiyama et al., 2019).

### Cytoskeletal organization and roles in asymmetric division

Asymmetric cell division (ACD) is a fundamental process in plant development, which occurs in two distinct phases: polarity establishment followed by division plane execution (Wen, 2020). Both phases rely on precise coordination of the cytoskeleton. In plants, the organization and function of cytoskeletal arrays during ACD have been studied in various model systems, including Arabidopsis zygotes, lateral root hairs, and stomatal development in both Arabidopsis and *Zea mays* (maize) (Facette et al., 2015; Kimata et al., 2016; Vilches Barro et al., 2019). These model systems have provided valuable insights into the roles of the cytoskeleton in polarity establishment during asymmetric cell division.

In *Arabidopsis thaliana*, zygotic development represents a critical transition from fertilization to embryonic patterning, with cytoskeletal dynamics playing pivotal roles in establishing the first asymmetric division. The zygote undergoes a characteristic elongation phase, during which cytoskeletal elements reorganize to define apical-basal polarity and division plane orientation (Kimata et al., 2016).

**FIGURE 3:**
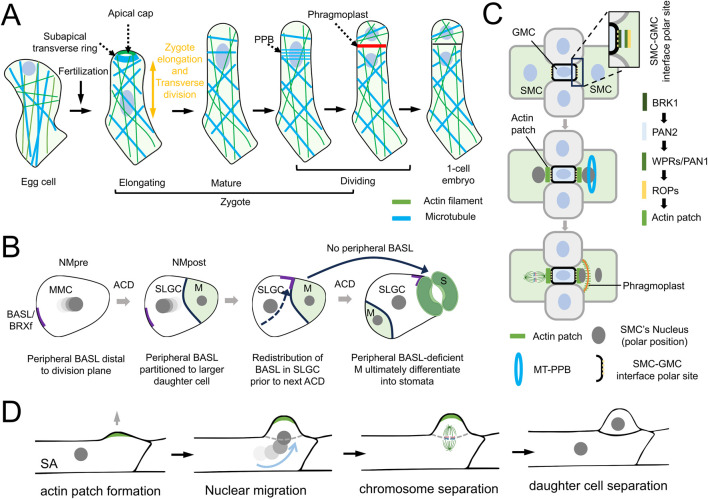
Dynamics of actin filaments and microtubules in asymmetric cell division. **(A)** Schematic of microtubules (MTs) and actin filaments (AFs) dynamics during zygote polarization. AFs are essential for nuclear positioning and division plane establishment, while MT ring demarcate the subapical domain and promotes apical-directed cell expansion. **(B)** Schematic of Arabidopsis stomatal lineage divisions. Two oppositely oriented nuclear movements, NMpre (pre-division) and NMpost (post-division), are shown during the asymmetric division of the stomatal meristemoid mother cell (MMC). The nuclear color darkens at later time points. Additionally, the polar localization of BREAKING OF ASYMMETRY IN THE STOMATAL LINEAGE (BASL) and BREVIS RADIX family (BRXf) plays a critical role in stomatal patterning.**(C)** Schematic of stomatal development in maize. In the asymmetric division of subsidiary mother cells (SMCs), the establishment of cellular polarity guides both the proper distribution of the cytoskeleton and the determination of asymmetric division orientation. **(D)** Schematic of asymmetric division in the protonemal subapical cell (SA) of the moss *Physcomitrium patens*. During preprophase/prophase, the SA assembles an apical actin patch (green) and protrudes the membrane to form a bulge (indicated by a gray arrow). The nucleus migrates into the bulge, followed by nuclear envelope disassembly and mitotic spindle formation.

During zygote elongation, F-actin forms two distinct configurations: an apical cap and longitudinal arrays in the apical domain (Figure 3A). Concurrently, MTs exhibit transverse alignment and organize into a subapical ring structure (Kimata et al., 2016; Webb and Gunning, 1991). Following elongation, the MT ring disassembles, and cortical MTs redistribute to form a preprophase band surrounding the nucleus (Figure 3A). Pharmacological and genetic analyses reveal that AFs are essential for nuclear positioning and division plane establishment, while the MT ring demarcates the subapical domain and promotes apical-directed cell expansion (Kimata et al., 2016).

Lateral root initiation in *A. thaliana* serves as a model for understanding asymmetric cell division (ACD). During this process, lateral root founder cells undergo asymmetric division to form a dome-shaped primordium. Prior to founder cell division, cortical microtubules (CMTs) in the central domain appeared isotropic in their organization, CMTs in the peripheral domain organized in transverse parallel arrays. In this process, MTs are necessary for the radial expansion of founder cells before the asymmetric division occurs. Additionally, the polar migration of the nucleus is dependent on the AFs network, which has been shown to regulate nuclear movement during asymmetric division (Vilches Barro et al., 2019). This suggests that AFs plays a key role in the polarized movement of the nucleus, a process likely conserved in plant cell polarity establishment.

In the stomatal lineage of Arabidopsis, asymmetric cell divisions (ACDs) play a vital role in establishing the proper stomatal pattern. ACD happens early in cell division when a mother cell divides asymmetrically, generating a larger cell (meristemoid, M) and a smaller cell (stomatal lineage ground cell, SLGC). In SLGCs, a cortical region lacking stable microtubules, regulated by polarity proteins like BASL/BRXf, is crucial for pre-mitotic nuclear migration (Figure 3B), positioning the nucleus away from the polarity crescent (Muroyama et al., 2020). In contrast, F-actin, in conjunction with its motor protein MYOXI-I, is necessary for post-mitotic nuclear migration, reorienting the nucleus toward the BASL/BRXf crescent in the SLGC and thereby reversing the cell’s polarity (Muroyama et al., 2020) (Figure 3B). These results highlight the essential roles of AFs and MTs in controlling the division orientation of SLGCs and consequently regulating stomatal development.

In *Zea mays* (maize), the stomatal complex is made up of four cells: a pair of guard cells (GCs) shaped like dumbbells, with two subsidiary cells (SCs) on either side. This structure forms through two asymmetric divisions followed by one symmetric division. The initial asymmetric division occurs in the young protodermal cells of the leaf, producing a small guard mother cell (GMC) and a larger pavement cell. Next, two adjacent subsidiary cell mother cells (SMCs) undergo asymmetric divisions, generating the SCs that surround the GMC (Figure 3C). Lastly, the GMC divides symmetrically to produce the two guard cells (Facette et al., 2015; Hepworth et al., 2018).

The mechanism of cell polarity during the first asymmetric division remains unknown. In contrast, the second round of asymmetric division, involving the SMCs, has established a clear pathway for cell polarity (Cartwright et al., 2009; Facette et al., 2019; Zhang et al., 2012). During the asymmetric division of SMCs, several polarized proteins accumulate in an orderly manner at the SMC-GMC interface to establish cell polarity (Figure 3C). The earliest identified polarized protein is BRK1, followed by two leucine-rich repeat receptor-like kinases (LRR-RLKs): PAN1 (Pangloss1) and PAN2 (Pangloss2), and then ROP GTPase (Figure 3C). Subsequently, cortical AFs accumulate at this site, forming a high-density structure known as the actin patch before the polarization of the premitotic SMC nucleus (Figure 3C). After SMC division, the actin patch is inherited by the SC and continues to enrich at the SC-GMC interface (Humphries et al., 2011).

Genetic studies on polarized protein knockout mutants (such as *brk1*, *pan1*, *pan2*, and *rop2/rop9*) have shown that the absence of actin patches at the interface between the SMC and GMC is associated with defective nuclear polarization and abnormal SMC division (Facette et al., 2015). These findings suggest that actin patches play a crucial role in SMC polarity establishment. However, the relationship between actin patches and nuclear polarization is not entirely deterministic. For example, the absence of actin patches during early SMC division does not always lead to nuclear mislocalization, highlighting incomplete penetrance of the phenotype. This partial penetrance suggests that the establishment of nuclear polarity likely involves a complex regulatory network, where actin patches contribute to, but do not solely dictate, the spatial cues necessary for proper nuclear positioning. Notably, In *Physcomitrium patens*, polarized actin patches act as cytoskeletal organizers to drive asymmetric division of protonemal subapical cells (SAs) (Figure 3D). Prior to division, spatiotemporally coordinated actin polymerization and Rho-of-Plant (ROP) signaling establish a cortical bulge, while concurrent nuclear migration from the cell center into this protrusion defines the division asymmetry (Figure 3D). This ROP-actin-nuclear axis underscores the evolutionary conservation of cytoskeletal machinery in plant cell polarity regulation (Yi and Goshima, 2020; Yi et al., 2025).

The arrangement and function of MTs in early SMCs, before nuclear polarization, remain unclear. Most studies have focused on the later stages of SMC asymmetric division, where MTs dominate the cytoskeletal structures, including the preprophase band (PPB), the mitotic spindle, and the phragmoplast (Figure 3C). These topics have been extensively reviewed (Facette et al., 2019; Lee et al., 2008; Lee and Liu, 2013; Livanos and Müller, 2019; Rasmussen and Bellinger, 2018; Rasmussen et al., 2011). Although the cytoskeleton plays important roles in stomatal formation, our understanding of its role in stomatal asymmetric division in dicotyledonous plants, such as Arabidopsis, and in grasses like maize, remains limited. Collectively, these findings indicate that the cytoskeleton is essential for stomatal formation, and the specific arrangement of cytoskeletal components provides an excellent system for studying cell polarity and cytoskeletal dynamics.

## Regulation of the cytoskeleton: the role of ROP and WAVE pathways

Plant cells establish polarity by integrating intrinsic and extrinsic signals to regulate actin filament and microtubule dynamics. Cell type-specific AF/MT architectures, essential for polarity and morphology, are primarily governed by currently known conserved pathways involving Rho-GTPases (ROPs), the SCAR/WAVE complex, and motor proteins. These regulatory mechanisms are evolutionarily conserved across diverse plant species and cell types.

### ROPs and ROP effectors

In plants, a plant-specific family of Rho-GTPases, known as ROPs, function as signaling switches (Craddock et al., 2012; Yang, 2008; Zheng and Yang, 2000). ROPs are critical regulators of cell polarity and act as upstream effectors of the cytoskeleton in various cell types.

In tip-growing cells, such as pollen tubes and root hairs, ROPs localize to specific plasma membrane domains (Li et al., 1998; Lin et al., 1996). For example, Arabidopsis ROP1 is activated and localized to the apical plasma membrane of pollen tubes (Figure 1A), where it regulates tip growth by activating two opposing ROP-interactive CRIB motif-containing (RIC) proteins. Specifically, RIC3 promotes F-actin disassembly by enhancing the formation of a Ca^2+^ gradient at the pollen tube tip, while RIC4 promotes F-actin assembly (Gu et al., 2005; Lee et al., 2008). Additionally, RIC1 in Arabidopsis localizes to the pollen tube tip, where it regulates AFs through its actin-binding, severing, and capping activities (Zhou et al., 2015).

Moreover, multiple ROP domains can coexist within a single cell to drive complex morphogenetic changes. For example, in xylem cells, ROP domains are required for proper secondary cell wall deposition. Active ROP domains at the secondary cell wall pit, where ROPGEF4 and ROPGAP3 activate ROP11, interact with MIDD1 to anchor it to the plasma membrane (Figure 2C). This interaction recruits Arabidopsis kinesin 13A, a microtubule depolymerizing protein, to the pit domain, leading to a microtubule-free zone that inhibits cell wall deposition (Oda and Fukuda, 2013). In contrast, MTs outside the pit domain are stabilized by MAP70. These ROP-activated domains regulate cortical microtubules, playing a key role in secondary cell wall deposition. Studies suggest that the ROP signaling pathway promotes the assembly of actin filament (AF) rings around the pit, regulating cell wall deposition. A pit boundary-localized protein, Wallin (WAL), interacts with F-actin to facilitate actin assembly at pit boundaries. WAL also interacts with BDR1 (Boundary of ROP Domain 1), which is recruited to the plasma membrane by ROP11 (Sugiyama et al., 2019). Recent studies have shown that FORMIN HOMOLOGY DOMAIN CONTAINING PROTEIN11 (FH11) is essential for AF ring formation, and misexpression of FH11 leads to rearrangements in ROP GTPase during xylem cell pit formation (Kijima et al., 2025). These findings suggest that the ROP pathway not only promotes AF accumulation at the pit boundary to enhance cell wall deposition but also forms a feedback loop, where AFs regulate the ROP pathway.

Additionally, pattern formation of pavement cell lobes is also regulated by the ROP pathway. ROP2 activates RIC4 to organize cortical actin microfilaments required for lobe growth, while it inactivates RIC1 to mediate microtubule rearrangement. This indicates that the ROP-RIC1-MT pathway promotes neck formation and antagonizes the ROP-RIC4-AF pathway by inhibiting ROP activation (Fu et al., 2005).

### WAVE-Arp2/3 pathway

The WAVE-Arp2/3 pathway is crucial for cytoskeleton organization and cell polarity. The Arp2/3 complex nucleates AFs and is activated by the WAVE complex (Zhang et al., 2013). The WAVE complex comprises five proteins: Sra1 (also known as Rac1-associated protein 1, CYFIP1, or Pir121), Nap1 (Nck-associated protein; also known as Nap125, Nckap1, Kette, or Hem2), Abi (Abl interactor), WAVE (Scar), and Brick1 (Brk1, HSPC300) (Szymanski, 2005). SCAR, a subunit of the WAVE complex, is the primary activator of Arp2/3, while BRK1 stabilizes SCAR (Le et al., 2006).

Interestingly, in trichomes, the WAVE-Arp2/3 pathway is recruited and activated by SPIKE, a member of the DOCK family of guanine nucleotide exchange factors (GEFs) (Basu et al., 2008). Additionally, microtubule (MT) rings play a role in ensuring that SPIKE is properly localized at the tips of trichome branches (Yanagisawa et al., 2018).

The WAVE pathway is essential for asymmetric division in SMC (Facette et al., 2015). In maize, two key subunits of the WAVE complex, BRK1 (the maize ortholog of Brk1/HSPC3000) and BRK3 (encoding NAP1), have been studied. Mutants in both genes exhibit defects in subsidiary cells and lack characteristic crenulations along the edges of pavement cells. Co-immunoprecipitation (Co-IP) followed by liquid chromatography-tandem mass spectrometry (LC-MS) assays confirmed that Brk1 and Brk3 co-express with other maize orthologs of WAVE complex subunits, highlighting the pathway’s role in SMC asymmetric division. Furthermore, genetic studies indicate that the BRK1-PAN1/PAN2-ROP GTPase pathway regulates actin patch formation at the interface between SMC and guard mother cells (GMC) (Facette et al., 2015; Humphries et al., 2011; Zhang et al., 2012). A recent study identified the WPR family of actin-binding proteins, which regulate actin patch formation prior to SMC division, suggesting that WPR functions downstream of the WAVE pathway (Figure 3C), opening new research avenues for exploring the connection between the cytoskeleton and cell polarity (Nan et al., 2022).

In moss protonemal cells, the WAVE-Arp2/3 pathway has also been studied in tip-growing cells. The maize ortholog PpBrk1 localizes to the cell apex, and the *Δbrk1* mutant exhibits reduced filament growth and disorganized actin arrays at the apex (Perroud and Quatrano, 2008). Similarly, in fucoid algae, the Arp2/3 complex regulates actin network organization at the rhizoid pole during zygote polarity establishment (Hable and Kropf, 2005). Overall, the WAVE-Arp2/3 pathway serves as a conserved mechanism for organizing cell polarity across various plant cell types, and though different downstream factors may regulate the cytoskeleton in specific contexts.

### Motor proteins

Motor proteins play a crucial role in regulating the cytoskeleton and maintaining cell polarity in plants, influencing both AFs and MTs. These motor proteins are involved in the formation of specialized structures within the cytoskeleton, establishing local polarity and regulating the development of distinct cell morphologies. This mode of regulation has been observed in only a few cell types so far. This section highlights studies on motor proteins in trichomes and moss protonemal cells.

In Arabidopsis, the KCBP protein functions as a bifunctional motor protein, interacting with both AFs and MTs through its MyTH4-FERM tandem domain (Tian et al., 2015). During trichome development, KCBP co-localizes with cortical MTs, especially at the apex and branching sites of growing trichomes. In the KCBP loss-of-function mutant (*kcbp-1*), the proper arrangement of transverse MT rings and the microtubule-depleted zone is disrupted. Additionally, the organization of cytoplasmic actin cables and the transverse cortical F-actin cap at the trichome apex is misaligned, resulting in defects in polarized branch elongation and tip growth.

In moss protonemal cells, two actin-based molecular motors, Myosin VIII and Myosin XI, play crucial roles in maintaining the apical cytoskeleton. Myosin XI co-localizes with apical AF clusters at the cell tip, facilitating and organizing actin polymerization (Vidali et al., 2010). Myosin VIII, another essential motor protein, is involved in cytoskeleton maintenance in tip-growing cells (Figure 1A). It overlaps with converging MTs and actin clusters near the apex. When Myosin VIII function is lost, the cytoskeletal networks fail to converge at the cell tip, indicating its vital role in MT convergence, potentially through the regulation of apical AFs by For2A, an actin nucleator from the formin II protein family. Furthermore, actin is necessary for Myosin VIII-mediated focusing of MTs, creating a positive feedback loop that sustains the cytoskeletal structure and function in tip-growing protonemal cells (Wu and Bezanilla, 2018). These findings underscore the importance of motor proteins in preserving the structural and functional integrity of the cytoskeleton in various cell types.

## Conclusion

Research on the cytoskeletal architecture in various plant species, such as moss *Physcomitrium patens*, grasses, Arabidopsis, and diverse cell types, has enhanced our understanding of cell polarity in plant development. Plant cell polarity is regulated by cell type-specific arrangements of AFs and MTs, coordinated by conserved molecular pathways. The cytoskeleton, along with its regulatory proteins, organizes polarity proteins and cellular components, including the nucleus, in a polarized manner. For example, in pollen tubes, AFs show distinct distributions and functions across different regions. In moss cells, both AFs and MTs localize to the apical region, while actin/Arp2/3 complexes form in trichomes, actin caps appear in zygotes, and actin patches are present in maize SMCs. These findings underscore the role of the cytoskeleton in establishing polarity.

Despite the diversity observed across species, some common principles in the regulation of cytoskeletal structures are emerging. Key regulatory proteins, such as ROP, WAVE, and motor proteins, are conserved across different species and cell types. Recent studies on specific cell types, including xylem cells, Arabidopsis and maize stomata, and rice pollen germination, have provided valuable insights into cytoskeletal organization and regulation in these contexts.

While significant progress has been made in understanding cytoskeletal roles in cell polarity, many questions remain. Recent advances in studying systems such as the first asymmetric division of the zygote (Kimata et al., 2016) and stomatal formation in the model plants Arabidopsis and maize (Nan et al., 2022) have demonstrated that the cytoskeleton is essential for cell polarity. However, the basic function of these cytoskeleton remains unclear, such as the role of actin patches in the asymmetric division of SMCs in maize, which exhibits polarized localization at the interface between the SMC-GMC. It is also important to gain a deeper understanding of how the cytoskeleton participates in the well-established stomatal development of Arabidopsis, in which the meristemoid mother cell (MMC) undergoes asymmetric division to establish cell polarity. Genetic tools, including a cell-type-specific “oryzalin” known as PHS DP and a “latrunculin” termed DeAct, have been successfully utilized to study cytoskeletal functions in root hair initiation by specifically disrupting AFs and MTs, respectively (Vilches Barro et al., 2019). Furthermore, progress in cytoskeletal research has been significantly enhanced by the discovery and utilization of appropriate cytoskeletal markers (e.g., Lifeact or ABD2-GFP), particularly in studies on cell polarity formation, which often focus on single cells. Overexpression of genes or the introduction of exogenous genes can impact single cell growth and differentiation, underscoring the need for the careful selection of minimally disruptive markers. For example, recent studies on the involvement of MTs in nuclear polarity movement in pollen tubes have tested various cytoskeletal-binding proteins or domain sequences, thereby enabling precise mapping of cytoskeletal distribution and dynamics (Wang et al., 2024). However, suitable markers are still lacking for some cell types. For instance, the dynamic arrangement of microfilaments during stomatal development in plants and the distribution of the cytoskeleton in the root apical meristem are not yet effectively marked. The development of cell-type-specific markers for AFs and MTs, combined with live-cell imaging technologies, would significantly advance research in this field.

Moreover, the regulatory factors controlling the cytoskeletal polarity distribution remain to be fully explored. The establishment of cell polarity is a dynamic process that integrates signal perception, transduction, and coordinated protein activity. This process generally requires a polarity initiation factor (e.g., PAN), a signaling mediator (e.g., ROPs), a cytoskeleton nucleation/branching/severing factor (e.g., SCAR/WAVE), and/or motor proteins. However, in some cell types, only the functions of certain elements have been revealed. For instance, in stomatal subsidiary mother cells (SMCs), the roles of ROPs and SCAR/WAVE in polarity initiation are well-characterized. Yet, the complete regulatory network—especially how endogenous signals or exogenous stimuli (e.g., hormonal cues) are sensed and translated into cytoskeletal reorganization—remains largely unexplored.

Additionally, while proteins such as ROP, WAVE, and motor proteins are relatively conserved across species and cell types, there may be species-specific or cell-type-specific regulatory pathways, particularly in zygotes, that govern cytoskeletal organization and cell polarity. A key challenge for researchers is to determine the subcellular localization of these proteins and to utilize relevant mutants in these specific single-cell types.

Notably, Liquid-Liquid Phase Separation (LLPS) has been suggested to play a critical role in establishing membrane polarity in animal cells (Liu et al., 2020). In plants, understanding how the cytoskeleton and polarity complexes interact with the plasma membrane to maintain polarity is essential, especially in cases where both the cytoskeleton and its regulators lack direct membrane-associated domains. Investigating the role of LLPS in plant cell polarity formation presents an exciting avenue for future research. Integrating these approaches with live-cell imaging offers a powerful strategy to study the roles of the cytoskeleton in plant cell polarity.
